# Adaptations to Postural Perturbations in Patients With Freezing of Gait

**DOI:** 10.3389/fneur.2018.00540

**Published:** 2018-07-17

**Authors:** Esther M. J. Bekkers, Sam Van Rossom, Elke Heremans, Kim Dockx, Surendar Devan, Sabine M. P. Verschueren, Alice Nieuwboer

**Affiliations:** ^1^Neuromotor Rehabilitation Research Group, Department of Rehabilitation Sciences, KU Leuven, Leuven, Belgium; ^2^Human Movement Biomechanics Research Group, Department of Movement Sciences, KU Leuven, Leuven, Belgium; ^3^Musculoskeletal Rehabilitation Research Group, Department of Rehabilitation Sciences, KU Leuven, Leuven, Belgium

**Keywords:** postural control, freezing of gait, Parkinson's disease, perturbations, falls, reactive postural control

## Abstract

**Introduction:** Freezing of gait (FOG) is a powerful determinant of falls in Parkinson's disease (PD). Automatic postural reactions serve as a protective strategy to prevent falling after perturbations. However, differences in automatic postural reactions between patients with and without FOG in response to perturbation are at present unclear. Therefore, the present study aimed to compare the response patterns and neuromuscular control between PD patients with and without FOG and healthy controls (HCs) after postural perturbations.

**Methods:** 28 PD patients (15 FOG+, 13 FOG−) and 22 HCs were included. Participants stood on a moveable platform while random perturbations were imposed. The first anterior platform translation was retained for analysis. Center of pressure (CoP) and center of mass (CoM) trajectories and trunk, knee and ankle angles were compared between the three groups using the Statistical Parametric Mapping technique, allowing to capture changes in time. In addition, muscle activation of lower leg muscles was measured using EMG.

**Results:** At baseline, FOG+ stood with more trunk flexion than HCs (*p* = 0.005), a result not found in FOG−. Following a perturbation, FOG+ reacted with increased trunk extension (*p* = 0.004) in comparison to HCs, a pattern not observed in FOG−. The CoM showed greater backward displacement in FOG− and FOG+ (*p* = 0.008, *p* = 0.027). Both FOG+ and FOG− showed increased co-activation of agonist and antagonist muscles compared to HCs (*p* = 0.010), with no differences between FOG+ and FOG−.

**Conclusions:** Automatic postural reactions after a sudden perturbation are similar between PD subgroups with and without FOG but different from HCs. Reactive postural control, largely regulated by brain stem centers, seems to be modulated by different mechanisms than those governing freezing of gait. Greater differences in initial stance position, enhanced by joint stiffening, could however underlie maladaptive postural responses and increase susceptibility for balance loss in FOG+ compared to FOG−.

## Introduction

When a stable posture is suddenly perturbed, the body must react and quickly adjust to recover balance. These reactive postural adjustments are considered to be automatic responses, since the activation onset of muscle contraction is shorter than voluntary reaction times (1). As a consequence of neuronal loss in the basal ganglia, patients with Parkinson's disease (PD) present with impaired motor automaticity particularly during voluntary repetitive sequential movements (2). However, continuous automatic motor control (allowing voluntary motor activity without conscious thought), may not necessarily cover automatic instantaneous postural responses as well. So far, studies on postural reactions to external perturbations showed delayed, inflexible and inefficient balance correcting responses in PD (3–6). The release of automatic postural responses involves brainstem structures, including the mesencephalic locomotor region, as well as basal ganglia input, particularly from the striatum (7–9). These regions are also known to be involved in freezing of gait (FOG) (10). People with FOG (FOG+) have larger impairments in central drive and movement automaticity than their non-freezing counterparts (11). The higher fall risk in this subgroup may possibly be explained by greater deficits in automatic postural control as well. In addition, larger impairments in reactive postural control may underlie the finding that when using clinical balance scales more severe balance deficiencies are apparent in FOG+ (12, 13).

The current state of the art on comparisons of responses to sudden balance perturbations in FOG+ vs. FOG− revealed that FOG+ had smaller balance corrective steps compared to FOG− (14). Hence, FOG+ seemed to either underestimate the size of the corrective step response needed to recover balance or tended to release responses with reduced gain. In contrast, two other studies investigating freezing-related balance control after sudden perturbations found no differences in Center of Mass (CoM) excursions or protective stepping behavior between groups with and without FOG (8, 15). However, FOG+ were less able to improve protective postural responses after repetitive perturbations (14, 15). Although the amplitudes of reactive postural responses and the quality of the first balance correcting step did not differ during forward platform translations, an attenuated response was found when providing a starting stimulus to accelerate responses in FOG+ (8). FOG+ showed delayed onset latencies and reduced acceleration responses of the tibialis anterior and rectus femoris compared to FOG− and healthy controls (8).

These previous studies mainly investigated reactive stepping, induced by compensatory behavior in response to perturbation. Until now, no study has compared the neuromuscular control of the lower limbs during perturbation between subgroups in PD, although earlier work showed attenuated agonist and increased antagonist activation and higher background activity in lower legs muscles in PD in comparison to controls (5, 6). FOG+ showed more flexed posture and altered electromyography (EMG)-patterns in the Tibialis Anterior (TA) and Medial Gastrocnemius (GM) during gait, especially in the gait cycles preceding freezing episodes (16, 17).

Unlike previous work, the current study aimed to unravel postural response differences between patients with and without FOG and healthy elderly, while a stable posture is maintained during perturbations. Compromised adaptive behavior in FOG was previously demonstrated during splitbelt treadmill perturbations during walking and suggested to be related to a reduced perception of locomotor asymmetry (18, 19). Adaptive behavior is time-varying and cannot fully be described by extracting one summary scalar at particular time points or regions of the motion trajectory. Therefore, in the current study we focused on postural adaptations during backward responses, which is the most common type of disequilibrium in aging and PD (5, 6, 20). We also chose to analyze our force plate and kinematic data using statistical parametric mapping (SPM) to capture extended trajectories of adaptive movement. We hypothesized that due to reduced automatic reactions, FOG+ would show greater instability in response to external perturbations compared to FOG− and HCs. In addition, EMG signals during postural reactions were compared between groups to unravel potential underlying abnormalities of neuromuscular control.

## Materials and methods

Twenty-nine Parkinson's disease and 22 healthy elderly adults (HCs) were included in the study. PD patients were categorized as FOG+ (*n* = 16) if they scored 1 on item 1 of the new Freezing of Gait Questionnaire (NFOG−Q) or FOG− (*n* = 13) if they scored 0 on this item. All participants met the inclusion criteria of a Mini-Mental State Examination (MMSE) > 23 and were able to stand upright for at least 15 min. In addition, inclusion criteria for the patient groups were: (i) diagnosis of PD based on the United Kingdom Parkinson's Disease Brain Bank Criteria (21), (ii) Hoehn and Yahr (H&Y) stage II or III (in ON-medication), and (iii) being on stable medication for the last month. Exclusion criteria comprised having a neurological disorder other than PD, vestibular disorders, musculoskeletal disorders or any other disease that could interfere with the experimental task. Patients were tested during the ON-medication state. The study was approved by the local ethical committee of the University Hospitals Leuven (s54665) and all participants provided written informed consent.

### Test protocol

#### Clinical assessment

Clinical assessments included demographic data, Montreal Cognitive Assessment (MoCA) and MMSE. The Mini Balance Evaluation System Test (Mini-BEST) was used to clinically assess postural control, including subdomain scoring (22). Disease severity in PD patients was determined by the Movement Disorder Society Unified Parkinson's Disease Rating Scale part III (MDS-UPDRS-III).

#### Data recording

The Computer Assisted Rehabilitation Environment (CAREN) movable platform operated with D-Flow software (Motek Forcelink, Amsterdam, The Netherlands) was used to randomly perturb posture to elicit postural responses. Three-dimensional marker trajectories were captured using 7 infrared cameras (Vicon, Oxford Metrics, UK, 100 Hz). Forty-five spherical reflective markers were placed on specific anatomic landmarks, using an adapted version of the Liverpool John Moores University model (23). Simultaneously, ground reaction forces were recorded using two force plates integrated in the CAREN platform (AMTI, Watertown, USA, 1,500 Hz). Lastly, surface EMG signals (Aurion, Zero-wire, IT, 1,500 Hz) of the tibialis anterior (TA) and gastrocnemius medialis (GM) were recorded during balance reactions (FOG+ = 15, FOG− = 12, HCs = 19). EMG data could not be sampled in all patients (*N* = 5) due to technical problems or due to not being able to lengthen the study in some cases. Electrodes were placed according to the SENIAM-guidelines.

Participants were asked to stand on the platform, looking straight ahead with arms crossed over the chest. Feet were slightly externally rotated at a standardized width of 15 cm between medial malleoli. Participants were instructed to keep their balance without moving their arms or taking a step as the platform moved, unless falling was imminent. All subjects wore a safety harness to prevent falling. The platform was accelerated by 1.5 m/s2, whereby the translation amplitude was standardized using the height of the participants as a scaling factor. Participants' posture was perturbed by random translations in four directions. The first anterior platform translations, imposing a backward balance perturbation, were retained for further analysis (24), as it was found earlier that this would provide the most revealing information on reactive postural control precluding learning effects.

### Data analysis

A customized bodybuilder kinematic model was used to determine 3D joint angles from the measured marker trajectories (Vicon BodyBuilder, Oxford Metrics). Ankle and knee angles were calculated as relative joint angles, expressing the relative angle between the two adjacent segments whereas the trunk angle was calculated as the angle relative to the vertical axis of the global reference frame. Ground reaction forces were first filtered using a 4th order low-pass Butterworth filter with a cut-off frequency of 10 Hz before calculating the combined Center of Pressure (CoP). Marker positions on the different anatomical landmarks were used to calculate the whole-body CoM, accounting for the relative mass of each individual segment. Patterns for SPM analyses were corrected to the initial position to account for different starting position. Peak values were extracted to indicate absolute position in space. The CoM and CoP were additionally corrected for platform movement. The extrapolated CoM (XCoM) was then calculated as measure of dynamic stability, according to the formula $XCoM=\;p+\frac{v}{\omega_{0}}$, where $\omega_{0}=\;\sqrt{gravity/vertical\;CoM\;position}$, *p* is the CoM position and *v* the initial CoM velocity (25). Raw EMG signals were first band-pass filtered using a 4th order Butterworth filter between 20 and 500 Hz, subsequently the signal was full-wave rectified. Lastly, a linear envelope of the signal was determined by applying a 4th order Butterworth low-pass filter with 40 Hz cut-off.

In analogy to previous studies, EMG signals measured from the TA and GM were quantified. Raw EMG signals were corrected by subtracting background activity, averaged of a period from 200 to 50 ms before perturbation onset (24, 26). EMG records were first normalized to maximum muscle activity over anterior and posterior perturbation conditions per subject. Magnitude of the corrected EMG signal was calculated by averaging over a time window 80–450 ms after perturbation onset (27). Muscle co-contraction index (CCI) of the GM-TA was calculated to determine antagonist/agonist activity ratio by applying the following calculation (28): CCI = 2^*^(EMG_ant_/EMG_ag_+EMG_ant_)^*^100. For the trials in which a protective step was necessary to maintain balance, step length and onset latency were calculated. Step length was calculated based on the heel marker position of the stepping leg. Step onset was determined as the time between perturbation onset and foot lift-off, identified from a vertical GRF lower than 10N. Stable, in-place responses were used for further posturography and EMG analysis. All variables were analyzed from 200 ms prior to 1,000 ms after perturbation onset.

### Statistical analysis

Statistical analysis was performed using IBM SPSS software (version 22). Demographic characteristics were compared between groups using ANOVAs, Kruskal-Wallis ANOVAs, independent sample *t*-test or Mann-Whitney *U*-tests, depending on the distribution of the variables. Categorical data were analyzed using Chi-square statistics. Abnormally distributed postural data were compared between groups using non-parametric Kruskal-Wallis ANOVA's and Chi-square for frequency data. Significant results were further investigated using a Mann-Whitney *U*-tests for *post-hoc* comparisons. Pattern analyses were performed using spm1d (non-parametric hypothesis testing) (v0.3) (www.spm1d.org). EMG measures were normally distributed and compared between groups using ANCOVAs. To account for differences in group characteristics, age was added as covariate. The critical threshold was set at *p* < 0.05, but Bonferroni-corrected for the post-hoc analysis.

## Results

### Demographics

Participants' characteristics are presented in Table 1. FOG+ and FOG− were well-matched for all demographic and disease-related parameters (*p* > 0.05). HCs however, were significantly older compared to FOG+ (*p* = 0.001) and FOG− (*p* = 0.012). Cognitive measures were similar between groups. Both FOG+ and FOG− showed more falls compared to HCs (*p* < 0.001; *p* = 0.031), but no subgroup differences were found. Scores on the Mini-BEST also differed significantly between HCs and PD subgroups (*p* < 0.001), but no dissimilarities were found between FOG+ and FOG−. Analysis of the subcomponents of the Mini-BEST revealed that the differences in the PD subgroups vs. HCs were most pronounced on reactive (*p* < 0.001) and dynamic (*p* < 0.001) postural control, with no apparent differences between FOG+ and FOG−. One patient with FOG was unable to complete the platform test and was excluded from data analysis.

**Table 1:** Participants' demographics.

	**HCs**	**FOG+**	**FOG−**	***p*-value**	***p*-value**	***p*-value**	***p*-value**
	***N* = 22**	***N* = 15**	***N* = 13**		**HCs vs. FOG+**	**HCs vs. FOG−**	**FOG+ vs. FOG−**
**Age** (years)	75.1 (9.1)	64.6 (8.3)	66.8 (10.4)	<**0.001**	**0.001**	**0.012**	0.735
**Gender** (M/F)	5/17	8/5	11/4	**0.005**	**0.006**	**0.033**	0.505
**MMSE** (0–30)	29.1 (1.6)	27.9 (1.1)	28.2 (1.7)	0.051	0.074	0.236	1.000
**MoCA** (0–30)	26.1 (2.9)	24.9 (3.1)	25.3 (3.9)	0.575	0.936	1.000	1.000
**Fall frequency** (6M)	0	3 (0–10)	2 (0–2.5)	<**0.001**	<**0.001**	**0.031**	0.306
**Mini-BEST** (0–28)	25.1 (1.9)	18.9 (3.4)	20.8 (4.3)	<**0.001**	<**0.001**	**0.001**	0.282
**H&Y stage** (1–5)		2 (2.1)	2 (2.1)	0.599			
**Disease duration** (years)		10.3 (6.9)	7.7 (6.5)	0.32			
**MDS-UPDRS-III** (0–132)		34.7 (11.3)	33.5 (13.9)	0.804			
**Disease dominance** (L/R)		8/7	9/4	0.390			
**LED** (mg/day)		545.2 (246.6)	517.9 (320.2)	0.802			
**NFOG-Q** (0–29)		13.8 (7.0)	0	<**0.001**			

*Means (± SD) or median (interquartile range 25–75%) are reported. P-values below the significance level of 0.05 are indicated in bold*.

HCs, Healthy controls; FOG+, patients with freezing of gait; FOG−, patients without freezing of gait; H&Y, Hoehn & Yahr; LED, Levodopa Equivalent Dosage; MDS-UPDRS-III, Movement Disorders Society Unified Parkinson's Disease Rating Scale part III; MMSE, Mini Mental State Examination; MoCA, Montreal Cognitive Assessment; NFOG-Q, New Freezing of Gait Questionnaire.

### Stepping responses

Stepping responses did not differ between groups. Percentage protective stepping responses were higher in both FOG+ (33.3%) and FOG− (30.8%) compared to HCs (22.7%), but did not differ significantly between these cohorts [X_(2)_ = 0.564, *p* = 0.754]. Average step lengths were smaller in both PD groups, but were not significantly different from HCs (FOG+: 25.27 ± 17.12 cm; FOG−: 24.89 ± 17.36 cm, HCs: 36.41 ± 18.26 cm; *p* = 0.589). Similarly, no differences were found in step latency between groups (FOG+: 385 ± 30 ms; FOG−: 314 ± 39 ms, HCs: 358 ± 50 ms; *p* = 0.141).

### Kinematics and kinetics during non-stepping postural response

#### Initial stance

During the time interval before platform translation onset (0–200 ms), significant differences between FOG+, FOG−, and HCs were found for both trunk (*p* = 0.01) and knee position (*p* = 0.01). FOG+ had a more stooped posture compared to HCs (trunk flexion: 19.61 ± 11.82 vs. 9.04 ± 4.69, *p* = 0.005), unlike FOG−. No differences were revealed between FOG+ and FOG−. Both PD subgroups had also more knee flexion (FOG+17.13 ± 10.23; FOG− 17.31 ± 9.28) compared to HCs (7.82 ± 6.87) (*p* = 0.005; *p* = 0.005).

#### Postural responses

##### SPM

**Figure 1:**
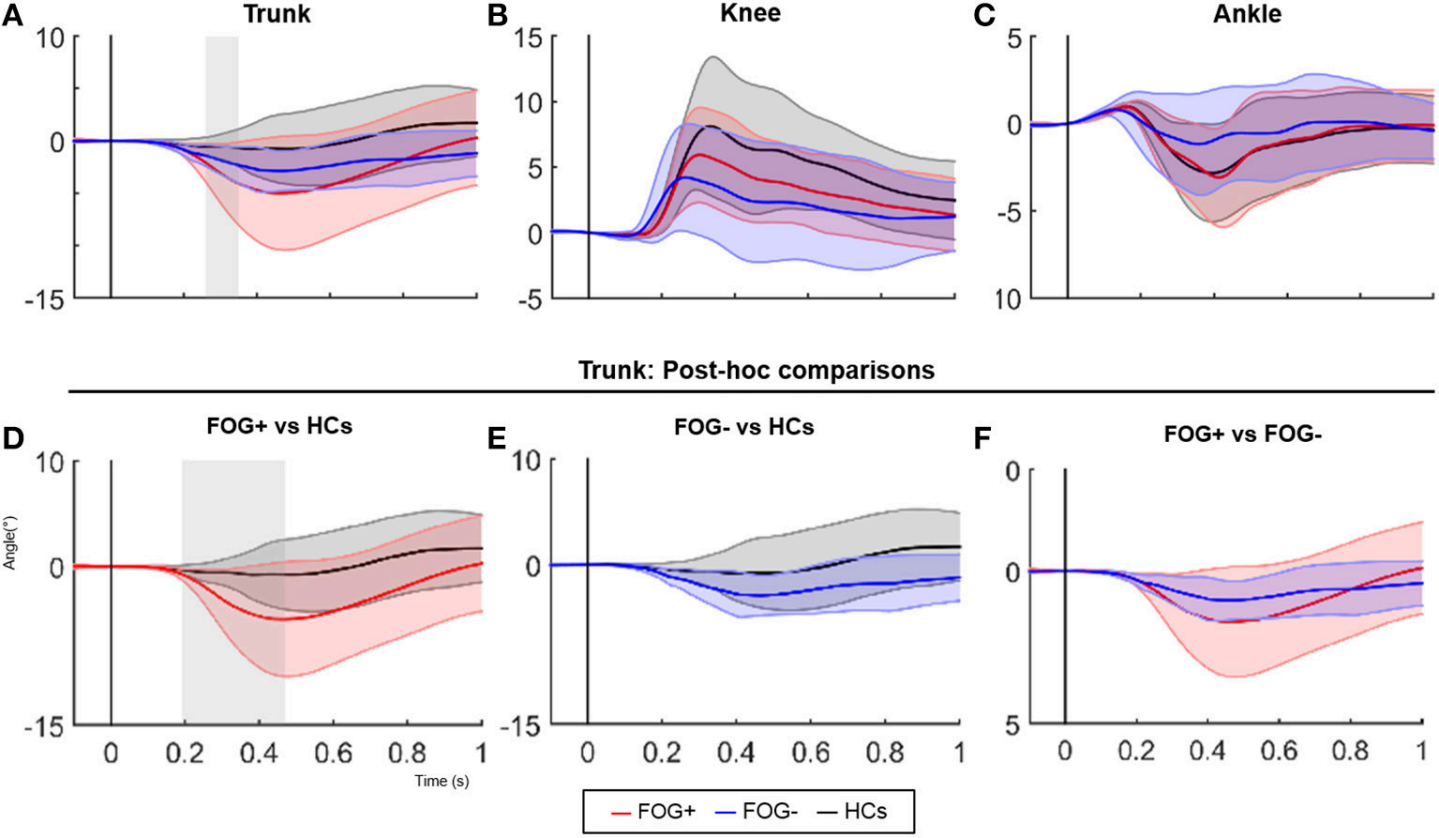
SPM analysis of joint angle responses following a posterior perturbation. Average (±SD) of angular displacement (°Δ) following a posterior perturbation in the three groups is shown in figure 1 (upper panels). Lower panels show post-hoc comparisons of the trunk pattern between groups. Gray zones indicate the time zones where groups significantly differ.

Kinematic data revealed a significant difference in trunk movement following a backward balance perturbation, this despite correcting for increased trunk flexion at baseline (significant between 267-368 msec following perturbation, *p* = 0.015) (Figure 1A). FOG+ responded with increased trunk extension compared to HCs between 184–554 ms after perturbation onset (*p* = 0.004) (Figure 1D), a pattern not observed in FOG− (Figure 1E). No differences were shown between FOG− and FOG+ (Figure 1F), probably due to higher variability in the FOG group. No significant differences between groups regarding knee and ankle angles were found (Figures 1B,C).

**Figure 2:**
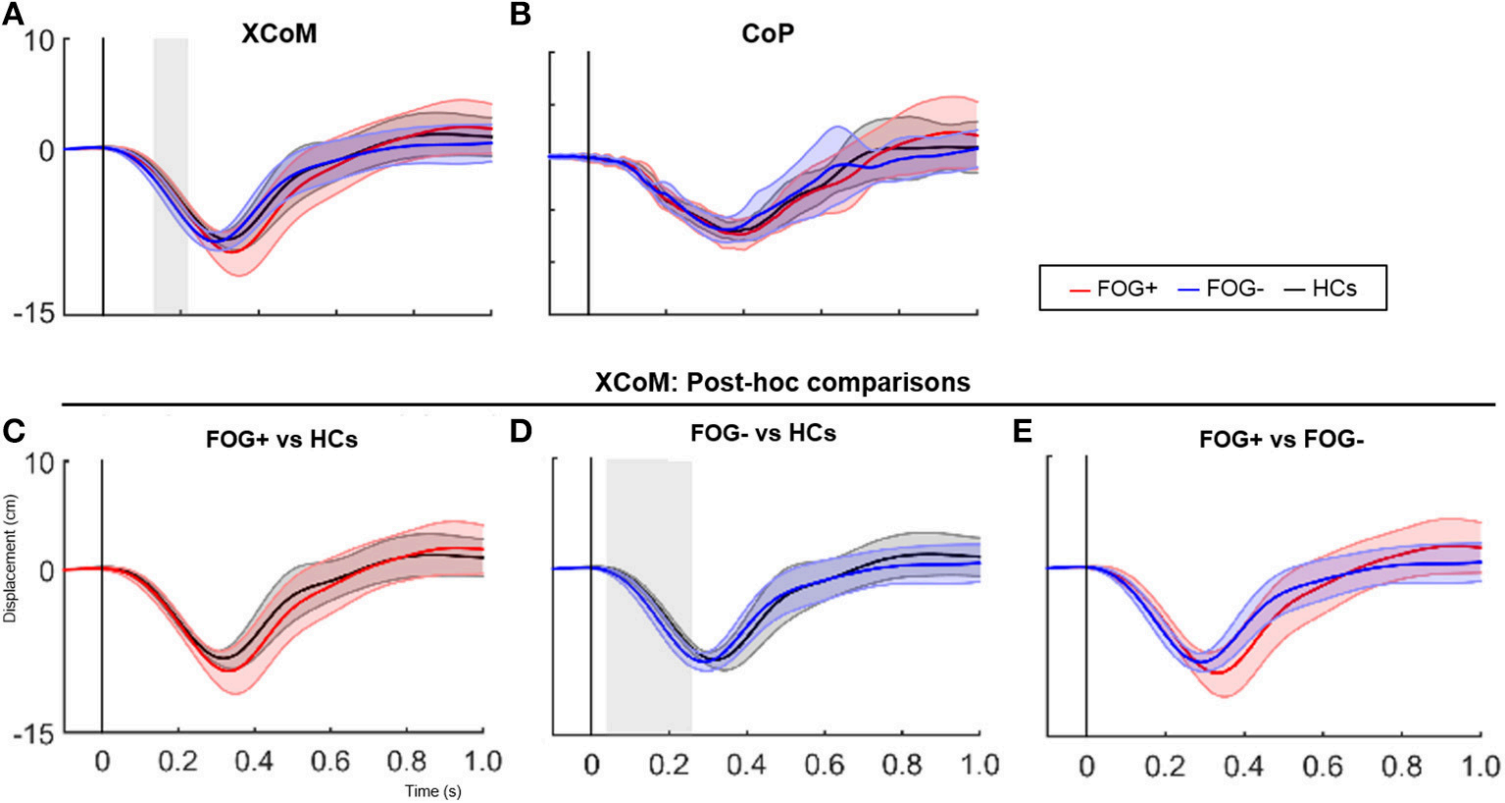
SPM analysis of XCoM and CoP responses following a posterior perturbation. Average (±SD) of XCoM and CoP displacement (cm) following a posterior perturbation in the three groups is shown in figure 2 (upper panels). Lower graphs show post-hoc comparisons between groups for XCoM. Gray zones indicate the time zones where groups significantly differ from each other.

Furthermore, XCoM responses differed significantly between groups immediately after perturbation onset (137–239 ms, *p* = 0.025) (Figure 2). Group comparisons revealed increased backward XCoM responses for FOG− compared to HCs at time interval 76–264 ms (*p* = 0.008) (Figure 2D). No differences were found between HCs and FOG+, although FOG+ tended to have greater backward CoM displacement during postural reactions especially at a later time zone (Figure 2C). In FOG+ more variable responses can be noted. FOG+ and FOG− did not differ regarding XCoM displacement, nor were group differences found for CoP.

Pooling the results of PD subgroups for the SPM analyses showed increased trunk extension between 115–492 ms (*p* = 0.004) and more backwards directed XCoM between 210–264 ms (*p* = 0.036) in PD vs. HCs (Supplementary Figure 1).

##### Peak values

Table 2 displays overall peak value differences between groups. Similar to the SPM analysis, greater trunk displacement was shown in FOG+ compared to HCs (*p* = 0.025). Absolute peak extension position was however not different between groups (*p* = 0.052), indicating that FOG+ moved not beyond the peak extension position of HCs. A similar pattern was found for knee angles, showing significant greater maximal knee flexion in FOG+ compared to HCs (*p* = 0.031), but the range of motion did not differ between groups. No differences in ankle joint angles and no differences compared to FOG− were found. Furthermore, larger XCoM displacement was seen in FOG+ compared to HCs (*p* = 0.027), but not in FOG−. XCoM time to peak and CoP parameters did not show any differences in postural response.

**Table 2:** Peak values of kinematic and kinetic data.

		**HCs**	**FOG+**	**FOG−**	***p*-value**	**HCs vs. FOG+**	**HCs vs. FOG−**	**FOG+ vs. FOG−**
Peak (°)	Trunk	6.88 (4.15)	14.21 (9.64)	8.05 (10.14)	0.052	0.055	1.000	0.276
	Knee	17.24 (4.61)	23.78 (7.87)	22.33 (7.23)	**0.027**	**0.031**	0.164	0.875
	Ankle	7.01 (4.95)	8.05 (4.63)	9.47 (5.57)	0.518	1.000	0.774	1.000
ROM (°Δ)	Trunk	4.12 (2.11)	7.35 (7.5)	4.01 (1.58)	**0.017**	**0.025**	1.000	0.064
	Knee	9.97 (4.80)	7.18 (3.78)	6.30 (3.54)	0.093	0.302	0.160	1.000
	Ankle	5.14 (2.31)	5.18 (2.61)	3.57 (1.82)	0.244	1.000	0.365	0.427
XCoM Peak (cm)		8.57 (0.10)	9.67 (1.8)	8.52 (0.70)	**0.023**	**0.027**	1.000	0.125
XCoM Time to Peak (s)		0.34 (0.03)	0.35 (0.03)	0.33 (0.02)	0.134	0.199	1.000	0.320
CoP Peak (cm)		7.46 (9.13)	7.68 (1.26)	7.39 (1.19)	0.668	1.000	1.000	1.000
CoP Time to Peak (s)		0.38(0.13)	0.36 (0.15)	0.34(0.11)	0.827	1.000	1.000	1.000

*Values represent means (+SD) of peak values and angular changes (°Δ) from initial stance position following perturbation. (ROM, range of motion; XCoM, extrapolated center of mass; CoP, center of pressure)*.

Pooled results showed significantly increased knee flexion in PD compared to HCs at maximal knee position (*p* = 0.007), but the total knee range of motion was smaller in PD (*p* = 0.031) (Supplementary Table 1). No other differences in angular responses were found. Also, CoP and XCoM parameters showed no differences between groups.

### Muscle responses

**Figure 3:**
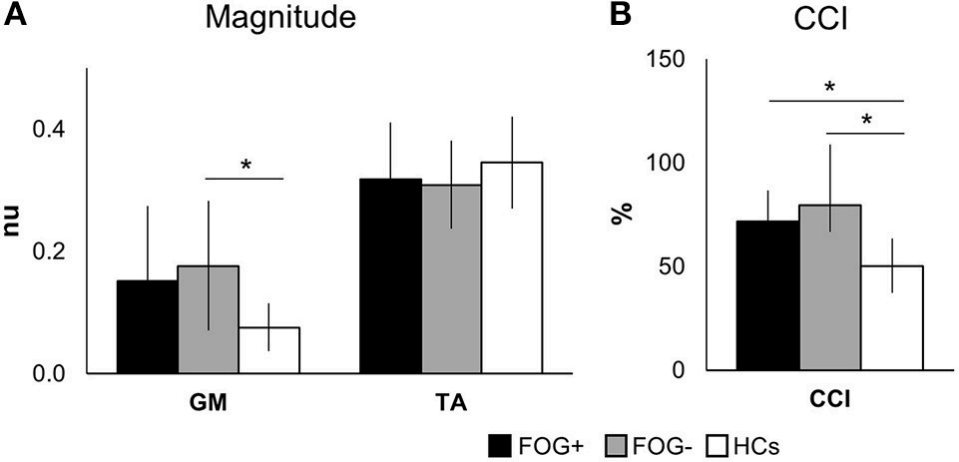
Mean EMG activity over 80–450 ms after perturbation onset. Graphs display group means ± SD for magnitude **(A)** and co-contraction of antagonists (GM) and agonists (TA), pooled for left and right leg **(B)**. *p < 0.05. (TA, tibialis anterior; GM, medial gastrocnemius; nu, normalized units; CCI, co-contraction index. *N* = 10 FOG+; *N* = 7 FOG−; *N* = 11 HCs).

Electromyography (EMG) responses are shown in Figure 3A. The magnitudes of the left and right signals did not differ between groups and were therefore pooled to increase power. Magnitudes of TA activation showed no differences between groups (*p* = 0.084). The EMG activity of GM muscles showed group differences (*p* = 0.039), with larger magnitudes in FOG− vs. HCs (*p* = 0.036), whereas FOG+ did not differ from both groups.

**Figure 4:**
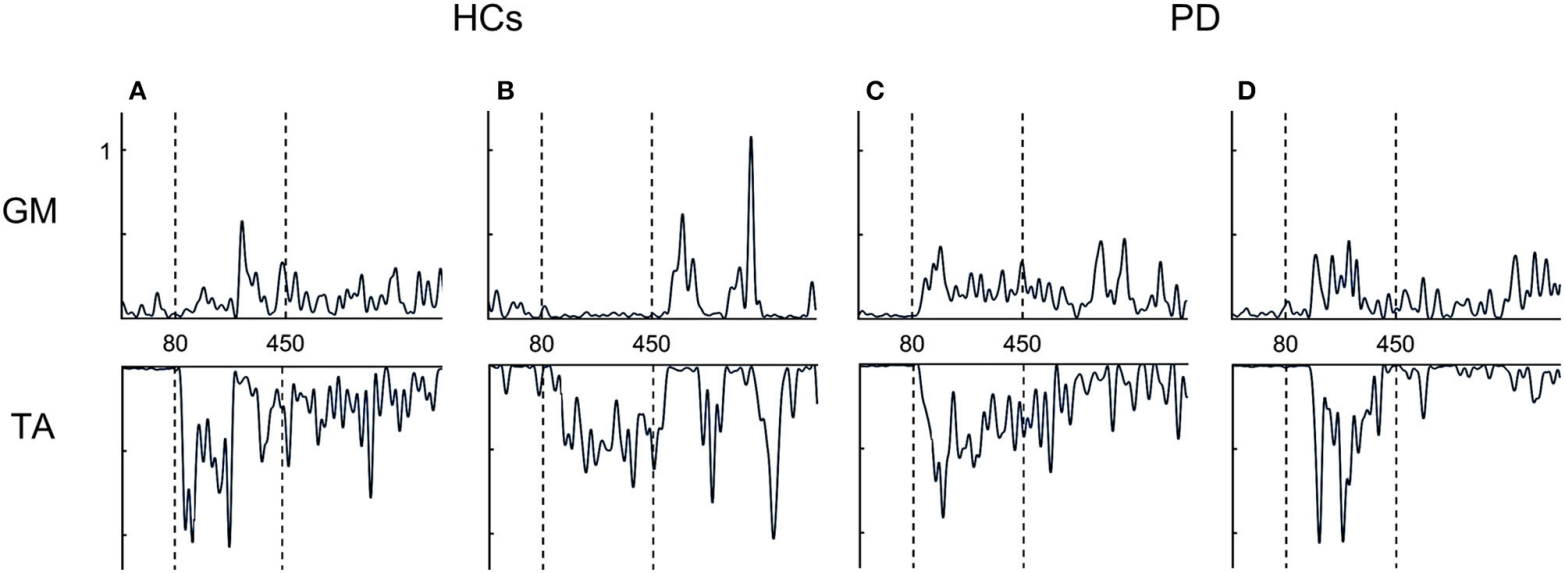
Examples of EMG profiles. HCs show alternating activation of agonists and antagonist **(A,B)**, whereas FOG+ and FOG− more often showed simultaneous activation of both agonist and antagonist (co-contraction) **(C,D)**. TA, tibialis anterior; GM, medial gastrocnemius.

Overall, both PD groups showed increased antagonist activity (GM) and reduced agonist activity (TA) in comparison to HCs. This was also illustrated by a significant increase in co-contraction (CCI) (*p* = 0.004) (Figure 3B). Compared to HCs, both FOG+ and FOG− showed increased co-contraction of lower leg muscles (*p* = 0.034, *p* = 0.005). Examples of these different activation patterns of agonist and antagonist are shown in Figure 4. No differences were found between FOG+ and FOG−. Analysis of the pooled data of PD subgroups showed increased GM activity (*p* = 0.012) compared to HCs, whereas no differences were found for TA. As a result, PD patients showed increased GM-TA co-contraction (*p* = 0.005) in response to a backward balance perturbation (Supplementary Figure 2).

## Discussion

This study aimed to compare postural adaptation patterns and neuromuscular control during backward balance responses in PD patients with vs. without FOG. Previous work using clinical balance assessments indicated that postural control is significantly worse in freezers and that this difference is most pronounced during reactive postural control in response to a leaning task (13). PD subgroups in the present study were well-matched for clinical profiles, including clinical balance outcomes and fall rates. Detailed posturographic and electromyographic data showed differences between FOG+ and HCs, which were not present in FOG−. However, no specific freezing-related differences were found. Abnormal trunk response patterns in freezers were demonstrated, revealing a larger degree of trunk extension directly following a backward balance perturbation in FOG+ compared to HCs. The XCoM results showed greater backward displacement in both FOG+ and FOG− than in HCs. Equally, EMG outcomes indicated increased co-activation of lower leg muscles, which were similar in both PD groups. Our results indicate that reactive postural control is largely comparable between subgroups with and without FOG and therefore our hypothesis was not confirmed. However, FOG+ have more pronounced abnormal reactive responses in the trunk when compared to HCs, and this when fall rate and balance capacity was equal in FOG+ and FOG−.

Both FOG+ and FOG−, who were able to counteract the balance perturbation with an in-place response, showed more retropulsive dyscontrol bringing subjects closer to their limits of stability, suggesting that PD patients were more unstable directly following the perturbation. Earlier research also showed increased backward instability in PD patients compared to HCs, irrespective of medication (4, 29) and a more backward shifted CoP position in FOG+ compared to FOG− and HCs during normal upright stance (30). Together, these findings explain the greater tendency for backward falls in PD (5, 20). The maximally extended position was however not different from HCs. Backward instability may be amplified by the increase in stooped position during initial stance, which enables to generate greater backward momentum when extending the trunk. Our results showed that FOG+ had an increased trunk and knee flexion, and reacted with exaggerated trunk extension following a perturbation. This could induce an even higher risk of backward falling in this subgroup. Earlier work suggested that stooping is a destabilizing factor, but does not solely explain postural instability in PD (31). As such, differences in initial joint position could trigger postural responses that increase susceptibility for balance loss. Whereas the XCoM was further backwards following the perturbation, as shown by both the SPM analysis and peak values in both FOG+ and FOG− compared to HCs, no differences in CoP were found. This can be explained by the fact that for stable responses, CoP, and CoM measures have a certain ceiling effect as these only vary within the limits of stability.

Patients with FOG+ did not differ in performing a corrective step to maintain balance compared to FOG− or HCs. This result is in line with those of Nonnekes et al., reporting no differences in stepping responses (8), although smaller steps in FOG+ compared to FOG− have also been reported (14). In addition, our findings support earlier research showing that switching to an alternative response strategy from feet-in-place trials to change-in-support, was similar between FOG+, FOG−, and HCs (14). This underscores that both patients with FOG+ and FOG− may not be more inflexible *per se*, but rather choose an inappropriate kinematic strategy to control their body. On the other hand, this study shows that the differences in kinematic responses between PD and HCs could be the result of inappropriate muscle coordination during a backward balance perturbation. At the muscular level, agonists should be activated in order to counteract for the backward loss of balance during a forward platform translation. Both FOG+ and FOG− showed increased antagonist activity of lower leg muscles (GM) in relation to reduced agonist activity (TA) compared to HCs. This led to increased co-activation of lower leg muscles in PD, which probably contributes to increased limb stiffness and less postural flexibility (1). Indeed, our data showed smaller ankle responses and a significantly reduced knee range of motion in PD vs. HCs. This stiffening of lower limbs could have resulted in a compensatory exaggerated trunk movement in PD, whereas HCs where able to respond from their knees. Therefore, stiffening of lower legs is seemingly also an explanatory factor for the typical retropulsion difficulties in PD. This finding is further supported by the fact that previous studies already showed earlier activation onset, increased magnitude of antagonist muscles and active stiffening in PD vs. HCs following a balance perturbation (5, 6). Although not examined directly, results of the present study confirm these findings and further extend these by demonstrating that PD subgroups with and without FOG show the same exaggerated co-activation during postural perturbations.

The basal ganglia play an important role in the regulation of muscle tone and coordination, mediated via reciprocal projections to the brainstem and cerebral areas. As such, they constitute fundamental structures for postural control (9, 32, 33). Whereas the basal ganglia-brainstem pathways contribute to automatic movement control and regulate muscle tone (32, 33), reactive postural responses are mainly mediated by brain stem centers *per se*, particularly the pontomedullary reticular formation (pmRF) and pedunculopontine nucleus (PPN) (7, 34). These notions come from studies investigating the StartReact phenomenon, in which responses are accelerated by triggering sensory stimuli. Hereby, it is assumed that some motor programs are subcortically stored in a prepared state and can be released quickly when triggered (7). The StartReact effect was shown to be attenuated in patients with FOG, but was intact in patients with postural instability (8). Additionally we showed that, in line with previous literature, automatic postural reactions are similar between PD subgroups. Reactive postural control therefore seems to affect different mechanisms than those governing FOG. However, this hypothesis remains speculative, was not directly assessed in the present study and requires further investigation.

The fact that slightly different results were found between groups on peak values vs. the pattern analyses, indicates that automatic postural reactions are also highly individualized. The individual nature of postural strategies was recently demonstrated in a study regarding anticipatory postural control, showing that anticipatory postural adjustments (APAs) prior to gait initiation differ between healthy people in general and that these differences are not related to aging (35), and possibly also not to disease. As such, postural differences may also be difficult to generalize to a population as a whole. Intriguingly, the variability in the FOG+ group was even more substantial. This highlights also the need for a more individualized approach in rehabilitation. For PD, exercise programs showed to improve kinematic measures and efficient muscle coordination in response to perturbations (27, 36). However future studies should confirm whether the effects for patients with FOG are similar as those for a general PD population. This is particularly of interest as FOG+ are known to have learning deficiencies that affect both motor and cognitive performance (14, 15, 37). This could therefore result in slower adaptation of muscle coordination. As such, FOG−related deficits are important to take into account to optimize rehabilitation programs for freezers.

Although this study provided more insights into the contributing factors to postural instability in PD, several limitations need to be taken into account. As indicated above, in postural control data, there is large variability, which hampers parametric statistics as well as the correction for confounding variables. This also points to the fact that strategies to maintain stability are dependent on individual strategies and therefore kinematic measures are difficult to use as reliable ‘predictors' of reactive postural control. Secondly, the elderly controls were significantly older than the PD patients, which may have resulted in an underestimation of our results. However, to counteract this effect, we added age as a covariate to our analyses. Furthermore, PD patients were measured while ON medication, which better reflects the real-life situation in which falls mainly occur, but can therefore not be generalized to the OFF state. In order to limit the confounding influence of medication, the PD subgroups with and without FOG were adequately matched. Moreover, dopaminergic medication may fail to improve postural impairment (4, 38, 39). Others indicated that medication has a highly variable impact on postural control (40). Testing in both ON and OFF state could, however, provide a more complete picture of freezing-related postural control, which would be valuable to assess in future studies. Further, we only measured EMG of the lower limbs. Based on our results, hip and trunk muscle responses may have provided additional information and could be of interest for further investigation. Lastly, the small sample size may have resulted in an underestimation of significant results and/or caused a lack of significant findings. EMG could not be sampled in all subjects and about 30% of our total participants reacted with a stepping response, not only limiting more detailed analysis of these patients' kinetic and kinematic measures but also could have biased the results. This should be taken into account for sample size calculations in future research.

To conclude, automatic postural reactions after a sudden perturbation are largely similar between subgroups of PD with and without FOG, but those with FOG have more abnormal responses compared to HCs. Postural response patterns were highly variable within groups and seem dependent on the individual. Differences in initial trunk and knee position, enhanced by joint stiffening, could trigger maladaptive postural behavior that predisposes patients to falling. Therefore, rehabilitation programs are important to improve efficient muscle responses and postural awareness, contributing to improved postural stability. In addition, present findings support personalized approaches for balance training in PD.

## Author contributions

KD, AN, and SV: Study design; EB, KD, and SD: Data acquisition. EB, SVR, SV, and AN: Data analysis and interpretation; EB and SVR: Drafting the article; EB, SVR, EH, SV, and AN: Article critical revision for important intellectual content; EB, SVR, EH, KD, SD, SV, and AN: Final approval of the submitted version.

### Conflict of interest statement

The authors declare that the research was conducted in the absence of any commercial or financial relationships that could be construed as a potential conflict of interest.

## Abbreviations

CCI: Co-Contraction Index
CoP: Center of pressure
FOG: Freezing of Gait
FOG+: Patients with FOG
FOG−: Patients without FOG
GM: Gastrocnemius Medialis
HCs: Healthy controls
PD: Parkinson's disease
SPM: Statistical Parametric Mapping
TA: Tibialis Anterior
(X)CoM: (Extrapolated) Center of mass.
