# Supplementary material for: Adaptations to Postural Perturbations in Patients With Freezing of Gait
Source: Front Neurol. 2018 Jul 17;9:540. doi: 10.3389/fneur.2018.00540 (PMC6056632; doi:10.3389/fneur.2018.00540)
Supplement: Supplementary file 1 [file Data_Sheet_1.docx]

Supplementary Material

Adaptations to postural perturbations in patients with freezing of gait

Esther M.J. Bekkers^1*^, Sam Van Rossom^2^, Elke Heremans^1^, Kim Dockx^1^, Surendar Devan^1^, Sabine Verschueren^3^, Alice Nieuwboer^1^

*** Correspondence:** Esther MJ Bekkers: [esther.bekkers@kuleuven.be](mailto:esther.bekkers@kuleuven.be)

# Supplementary Figures and Tables

## Supplementary Figures


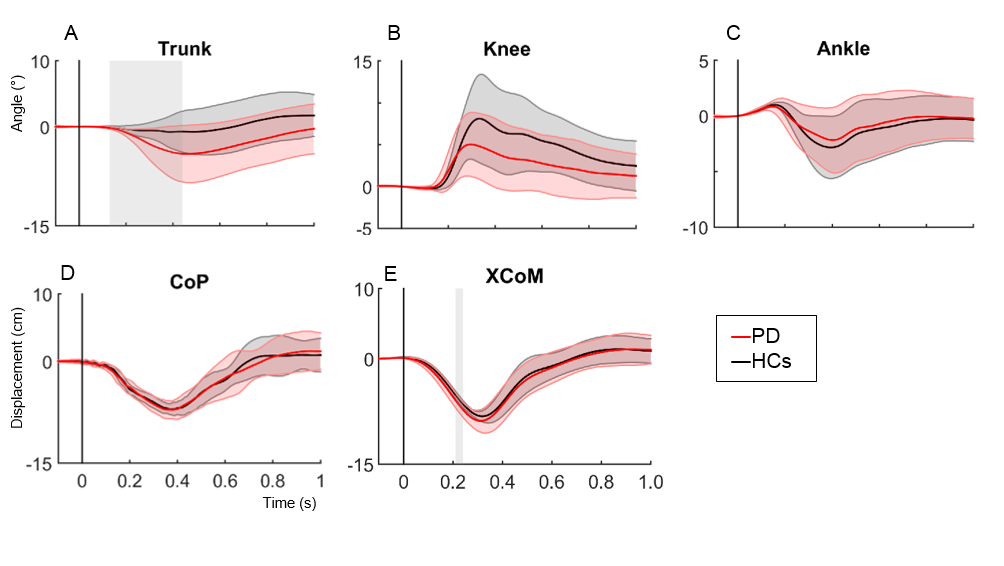


**Supplementary Figure 1 - SPM analysis of XCoM and CoP responses in PD and HCs following a posterior perturbation.** Figure shows the patterns of average (+SD) angular (°), XCoM and CoP displacement (cm) following a posterior perturbation in PD and HCs. Grey zones indicate the time zones where groups significantly differ from each other.

**
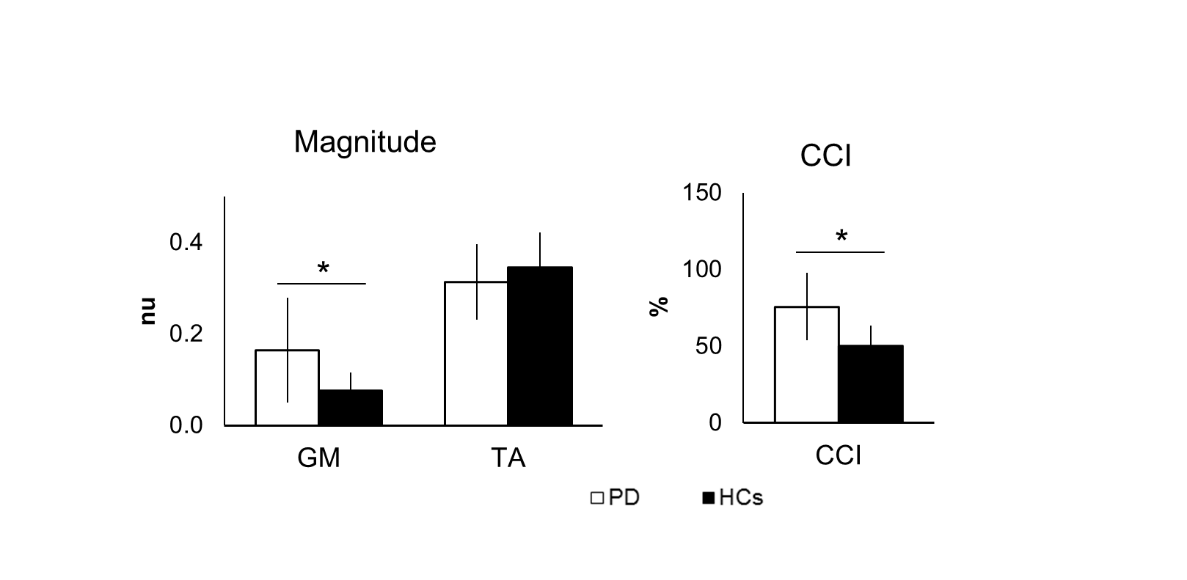
**

**Supplementary Figure 2 – Mean EMG activity over 80-450ms after perturbation onset.** Graphs display group means ± SD for magnitude (upper panel) and co-contraction of antagonists (GM) and agonists (TA), pooled for left and right leg. *p<0.05. (TA, tibialis anterior; GM, medial gastrocnemius; nu, normalized units; CCI, co-contraction index. N=17 PD; N=11 HCs).

## Supplementary Tables

|  |  | **HCs** | **PD** | ***p*-value** |
| --- | --- | --- | --- | --- |
| Peak (°) | Trunk | 6.88 (4.15) | 11.61 (10.07) | 0.072 |
|  | Knee | 17.24 (4.61) | 23.16 (7.44) | **0.007** |
|  | Ankle | 7.01 (4.95) | 8.65 (4.95) | 0.328 |
| ROM (°Δ) | Trunk | 4.12 (2.11) | 5.94 (3.89) | 0.096 |
|  | Knee | 9.97 (4.80) | 6.81 (3.61) | **0.031** |
|  | Ankle | 5.14 (2.31) | 4.50 (2.39) | 0.421 |
|  |  |  |  |  |
| XCoM Peak (cm Δ) |  | 8.57 (0.10) | 9.19 (0.15) | 0.056 |
| XCoM Time to Peak (s) |  | 0.34 (0.03) | 0.34 (0.2) | 0.236 |
| CoP Peak (cm Δ) |  | 7.46 (9.13) | 7.49 (11.84) | 0.936 |
| CoP Time to Peak (s) |  | 0.38 (0.13) | 0.35 (0.14) | 0.587 |

**Supplementary Tabel 1 - Peak values of kinematic and kinetic data in PD and HCs.** Values represent means (+std) of peak values and angular changes (°Δ ) from initial stance position following perturbation. (ROM, range of motion, XcoM, extrapolated center of mass).
